# Effects of nature-based mindfulness training on resilience/symptom load in professionals with high work-related stress-levels: findings from the WIN-Study

**DOI:** 10.1108/MIJ-10-2019-0001

**Published:** 2019-11-04

**Authors:** Caroline Lücke, Sylvia Braumandl, Bernhard Becker, Sebastian Moeller, Christina Custal, Alexandra Philipsen, Helge H.O. Müller

**Affiliations:** Department of Psychiatry and Psychotherapy, University Hospital Bonn, Bonn, Germany; GrenZENlos – Naturseminare, Neukirchen vorm Wald, Germany; Comes Unternehmensberatung, Oldenburg, Germany; Department of Psychiatry and Psychotherapy, University Hospital Bonn, Bonn, Germany; Department of Psychiatry and Psychotherapy, Friedrich-Alexander University Erlangen-Nuremberg, Nuremberg, Germany; Department of Psychiatry and Psychotherapy, University Hospital Bonn, Bonn, Germany

**Keywords:** Mindfulness, Intervention, Work-related stress, Nature-based mindfulness training, Stress prevention

## Abstract

**Purpose:**

The levels of work-related stress and the incidence rates of subsequent related illnesses are increasing in our society, leading to high individual and socioeconomic burdens. Mindfulness training has been shown to be an effective method of improving stress resilience. This paper aims to investigate the efficacy of nature-based mindfulness training in professionals with high levels of work-related stress.

**Design/methodology/approach:**

In this controlled pilot study, a total of 56 volunteers completed a nature-based mindfulness training progam and were compared to 8 participants (waitlist controls). Psychometric assessments were performed at baseline and after two and four months of training.

**Findings:**

After two months of training, the scores for self-efficacy, sense of coherence, level of mindfulness and overall psychiatric symptom load had significantly improved in the intervention group, while the control group did not show any significant improvements. A comparison between the intervention and control groups showed a significant difference regarding the sense of coherence only.

**Research limitations/implications:**

Since this was an exploratory study with a small control group, further studies are needed to verify our findings.

**Practical implications:**

In conclusion, nature-based mindfulness training seems to be a promising tool for the improvement of resilience and overall psychological health in professionals.

**Originality/value:**

This was the first study to systematically investigate effects of nature-based mindfulness training in people with high work-related stress.

## Introduction

Mindfulness is a psychological technique intended to focus attention on the present moment without judgment. Originating in Asian philosophy and Buddhist traditions (Greco and Hayes, 2011), the approach was made available and popularized in the Western world in the form of “mindfulness-based stress reduction” (MBSR), a systematic program developed by Kabat-Zinn (2010). The therapeutic approach consists of a combination of breathing exercises, meditation, mindful body perception (body scan) and yoga elements. The exercises are intended to train participants in the awareness and acceptance of the presence and changing of sensual impressions, physical sensations, thoughts and emotions in each moment (Kabat-Zinn, 2006).

The efficacy of the systematic MBSR program and mindfulness mediation with regard to stress reduction has been shown in several studies (Khoury *et al.*, 2015). Further studies showed that mindfulness training reduces the symptoms of depression and anxiety (Hofmann *et al.*, 2010), improves self-esteem and self-regulating behavior and helps practitioners cultivate a generally positive attitude toward life (Brown and Ryan, 2003). A meta-analysis showed that MBSR had a favorable effect on the outcomes of chronic conditions (Bohlmeijer *et al.*, 2010). Mindfulness-based approaches are not exclusively used in psychotherapeutic settings but are increasingly incorporated into preventive programs, including in the context of work-related health and satisfaction (Günthner and Batra, 2012). In recent years, there has been an increase in the number of workdays missed by employees because of psychiatric symptoms, as shown by data from major German health insurance providers (Techniker Krankenkasse, 2016). In this context, the condition referred to as “burnout,” which mainly consists of symptoms of depression, anxiety and exhaustion due to work-related stress, has been increasingly diagnosed by physicians and is controversially discussed in health politics and the public sphere.

While work-related stress factors such as a high workload, long or irregular working hours and tight timelines often cannot be easily changed by employees, stress-affected professionals may nevertheless reduce their individual burdens by improving their psychological resilience with regard to the effects of those stressors. In this context, the concepts of “self-efficacy” and “sense of coherence” are important, as both are connected to an improvement in the subjectively perceived psychosocial burden (Lazarus and Folkman, 1984; Bengel *et al.*, 2002). Mindfulness training has been shown in studies to improve both the perceived level of self-efficacy and the sense of coherence (Chang *et al.*, 2004; Soysa and Wilcomb, 2015; Ando *et al.*, 2011).

The present pilot study was conducted to investigate the efficacy of nature-based mindfulness training with regard to the perception of work-related stress by people who have a high level of work-related pressure. The structured program presented herein combined mindfulness training with the health-promoting elements of outdoor experiences. In a meta-analysis, favorable effects of nature-based activities such as walking, hiking and outdoor meditation or yoga have been shown (Barton and Pretty, 2010). Thus far, the combined effects of the elements of mindfulness training and the experience of nature have not been systematically investigated. In comparison to classic programs, such as MBSR, the nature-based approach may have an advantage because it offers the additional physiological and psychological benefits of outdoor activities. Furthermore, the outdoor setting may seem particularly attractive to some individuals, potentially resulting in better compliance and continuation rates.

## Methods

### Study design

Potential participants were recruited by the study team within their direct work environment. Most participants worked in fields with a high general stress level, such as the police, health care professionals and caregivers, but individuals reporting high levels of work-related stress who worked in other fields were eligible to participate as well. The exclusion criteria were current psychiatric conditions, current psychopharmacological or psychotherapeutic treatment, previous participation in mindfulness programs and alcohol or drug abuse.

After the participants received detailed information about the study and provided written informed consent, baseline psychometric measurements were obtained. The majority of the participants started nature-based mindfulness training shortly after the baseline measurements were taken, while some participants were randomly allocated to the control group. Participants in the control group had an intervention-free waiting time of two months with psychometric measures taken at the end of the waiting time (=T2). Later, they started nature-based mindfulness training that was analogous to that provided to the intervention group.

Nature-based mindfulness training was performed according to a structured training course certified by the verifying authority for prevention courses of the German health insurance agencies. The training consisted of two evening sessions per week and one half-day intensive training per month plus homework exercises. The training was performed over a four-month period. Psychometric measurements were obtained after two months (T2) and after four months (T3). All training sessions were performed by the same therapist (SB) to avoid rater bias.

The study was approved by the local ethics committee of the Carl von Ossietzky University in Oldenburg, Germany before the training was initiated.

### Psychometric outcome measures

The self-rated questionnaire [“Skala zur Allgemeinen Selbstwirksamkeit (*SWE*),” Schwarzer and Jerusalem (1999)] measures the perception of self-efficacy. Based on the concept of self-efficacy proposed by Bandura (1977), the 12 items assess the proband’s expectations of constructively solving new tasks and mastering difficulties. The SWE total score indicates the overall level of perceived self-efficacy.

The sense of coherence scale, nine-item short form (*SOC-L9*) is a self-rated instrument to measure the sense of coherence according to the concept proposed by Antonovsky (1979). A strong sense of coherence is associated with dispositional personal coping abilities and generalized resistance resources. The subscores SOC S and SOC G are measurements that provide individual grades according to the participant’s sense of coherence.

The Freiburg mindfulness inventory [*FMI* (Walach *et al.*, 2006), 14-item short version] is a validated, self-rated questionnaire constructed to evaluate therapeutic interventions focusing on mindfulness. It assesses a person’s level of mindfulness in everyday life, including the aspects of attention, acceptance and mental openness. The summed score referred to as FMI G (sum of items 1-12 and 14) is a measure of the overall level of mindfulness.

The symptom checklist 90 (*SCL-90*) is a widely used tool that measures the psychological and psychiatric symptom loads in various dimensions of psychopathology. The SCL global severity index (SCL GSI) is calculated by the sum of the 90 items of the SCL divided by the number of answers. It indicates the overall psychiatric symptom load.

### Statistical analysis

For the statistical analysis, only subjects who completed the study and provided data for the Timepoints T1-T3 were included. After the exclusion of drop-outs and subjects with incomplete data records, the analysis set comprised 56 patients in the intervention group and 8 subjects in the control group.

Data analysis was performed with GraphPad software (San Diego, CA).

The data analysis was performed as pre-post comparisons between the baseline (Timepoint 1) and the posttreatment Timepoints 2 and 3, using paired *t*-tests. In addition, score differences between T1 and T2 were compared between the treatment and control groups using unpaired *t*-tests. Because this was an exploratory pilot study, multiple testing was performed, and the *p*-values need to be interpreted with great care. Furthermore, the generalizability of the results of the between-groups comparisons is strongly limited by the small number of subjects in the control group.

The following outcome parameters were included in the statistical analysis and tested for significant pre-post and between-group differences: SWE sum, SOC S, SOC G, FMI G and SCL GSI (sum/90).

## Results

### Study population

A total of 107 participants in the intervention group and 12 participants in the control group provided baseline data. A considerable proportion of patients did not proceed with the study after the baseline or had to be excluded from the analysis due to insufficient psychometric data. For 56 participants in the intervention group and 8 participants in the control group, psychometric data at the baseline (T1) and after two months of training (control group: intervention-free waiting) were available, and these participants were included in the analysis. In the intervention group, 42 participants (75 per cent) were female and 14 participants were male; in the control group, 6 patients (75 per cent) were female and 2 patients were male. The mean age at the study start was 51.3 years in the intervention group and 40.75 years in the control group.

### Pre-post comparisons

**Figure 1:**
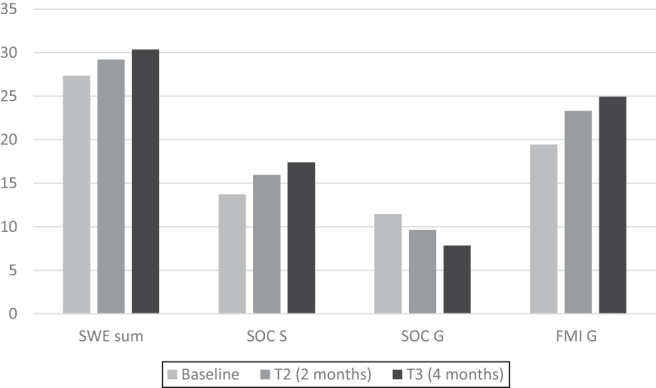
Development of self efficacy (SWE sum), sense of coherence (SOC S, SOC G) and mindfulness (FMI G) in the intervention group under nature-based mindfulness training

In the intervention group, the outcome scores showed improvements in self-efficacy, mindfulness, sense of coherence and overall mental health over the course of the study. Between the baseline (T1) and after two months of training (T2), the scores for the SWE sum, SOC S and FMI G (all indicating better outcomes) significantly increased, while the scores for SOC G and SCL GSI (indicating worse outcomes) decreased (Table I, Figure 1). As Table I and Figure 1 shows, there were further subtle score improvements toward the end of the study (T3, four months).

In the control group, there was a statistically significant increase in the SOC G score, indicating a worsening of these participants’ sense of coherence between T1 and T2, while comparisons for the other outcome parameters did not show any significant pre-post differences (Table I).

### Between-group comparisons

To test for differences between the intervention and control groups, the changes in scores from T1 to T2 were compared. There was a statistically significant difference between the two groups for the SOC S score (*p* = 0.0113) and the SOC G score (*p* = 0.0065) and a trend toward significance for the SCL GSI score (*p* = 0.0064), while the differences in the SWE sum score (*p* = 0.64) and the FFA G score (*p* = 0.42) were not statistically significant. As described above, these exploratory results need to be interpreted carefully because of the small number of controls and the performance of multiple testing.

## Discussion

This study was the first to systematically assess the effects of nature-based mindfulness training on psychological outcomes in professionals with high work-related stress levels. Our data show that the participants indeed profited from the training: as expected, the level of mindfulness, the sense of self-efficacy and the sense of coherence significantly increased. Interestingly, while the score improvements in these outcome dimensions were comparably mild, the overall psychiatric burden as measured by the SCL-90 decreased substantially, i.e. by 40 per cent. This may indicate an additive effect of the targets of mindfulness training, such as self-efficacy, a sense of coherence, self-acceptance and relaxation, on a person’s overall psychological stability. Furthermore, it may imply that nature-based mindfulness training has additional beneficial effects that were not directly assessed by these more specific psychometrical instruments. Factors that may play a role in the beneficial effect of nature-based mindfulness training could be the direct consequences of physical activity and the experience of nature, which are known to have mood-stabilizing effects (Blake, 2012; Barton and Pretty, 2010), as well as the social aspects of participating in a group activity and the regularity of the training.

The comparison of the outcomes of the intervention group with those of the waiting control group eliminates the possible biasing of the results by spontaneous improvement. However, a statistically significant difference between groups was only found for the sense of coherence scores; therefore, the data need to be interpreted with great care. However, because the control group in this study was very small, and the data showed a lack of improvement in the control group, a statistically significant difference between groups may be shown in future studies with larger sample sizes.

A further interesting point to be investigated in future studies may be the sustainability of the effects after the end of regular, structured training. Because the program was intended to train participants to use techniques that can be applied regularly in their daily lives, there seems to be a good chance that the participants can profit from the training in the long term. However, factors such as regularity and the level of commitment to the training may be more difficult for participants to sustain after the end of structured training and for some patients, the loss of the group experience may hinder the maintenance of positive effects.

In conclusion, nature-based mindfulness training seems to be a promising tool for the improvement of resilience and overall psychological health in people with high levels of work-related stress.

## Acknowledgements

*Authors’ contributions*: CL performed the statistical analyses and wrote the paper. HHOM, BB and SB conceived of the study, and HHOM is the senior author. SB performed the trainings, while SM, CC and AP gave their expert opinions on the mindfulness-based approaches. All authors were directly involved in writing the drafts of the manuscript and approved the final version of this manuscript.

*Funding*: This study was partially funded by the charitable foundation VR Stiftung.

*Registration*: This study was preliminarily registered by the local ethics committee of the Carl von Ossietzky University in Oldenburg, Germany and has been registered with the general health insurance agencies in Germany (Zentrale Prüfstelle Prävention gesetzlicher Krankenkassen ID: 20190517-S10175).

Conflicts of interest: All authors report that they have no conflicts of interest.

## Figures and Tables

**Table I:** Psychometric sum scores of participants in the invention group and in the control group over the course of the study

	SWE sum	SOC S	SOC G	FMI G	SCL GSI
***Intervention group***					
**T1 (baseline)**	27.36	13.71	11.48	19.45	0.99
**T2 (2 months)**	29.21	15.96	9.66	23.30	0.68
**T3 (4 months)**	30.36	17.39	7.84	24.95	0.59
***p*-value T1 vs T2**	<0.0001	0.0003	0.0018	<0.0001	<0.0001
***Control group***					
**T1 (baseline)**	28.13	15.75	8.00	22.25	0.79
**T2 (2 months)**	29.5	13.75	10.38	24.75	0.78
***p*-value T1 vs T2**	0.1112	0.1382	0.0013	0.1553	0.9648
